# Different patterns of myocardial iron overload by T2* Cardiovascular MR as markers of risk for cardiac complication in thalassemia major

**DOI:** 10.1186/1532-429X-14-S1-M1

**Published:** 2012-02-01

**Authors:** Antonella Meloni, Vincenzo Positano, Petra Keilberg, Brunella Favilli, Claudio Ascioti, Gianluca Valeri, Angelo Zuccarelli, Anna Pietrapertosa, Massimo Lombardi, Alessia Pepe

**Affiliations:** 1 CMR Unit, Fondazione G.Monasterio CNR-Regione Toscana and Institute of Clinical Physiology, Pisa, Italy; 2 Struttura Complessa di Cardioradiologia-UTIC, P.O. “Giovanni Paolo II”, Lamezia Terme, Italy; 3 Dipartimento di Radiologia, Azienda Ospedaliero-Universitaria Ospedali Riuniti "Umberto I-Lancisi-Salesi", Ancona, Italy; 4 Centro trasfusionale e di microcitemia, Ospedale civile, Olbia, Italy; 5 Servizio Regionale Talassemie, Policlinico, Bari, Italy

## Background

Cardiac complications mainly related to myocardial iron overload (MIO) remain the main cause of morbidity and mortality in thalassemia major (TM). Thalassemia cardiomyopathy is treatable and partly reversible if appropriate chelation therapy is instituted in time. The validated multislice multiecho T2* Cardiovascular Magnetic Resonance (CMR) technique has permitted to quantify segmental and global myocardial iron burden detecting different patterns of iron overload. Aim of our study was to verify the risk of cardiac complications related to different patterns of MIO in a large cohort of TM patients.

## Methods

We considered 812 TM patients for who CMR and cardiac data were collected in a central data base. Three short-axis views (basal, medium, apical) of the left ventricle were acquired using a multislice multiecho T2* sequence. Using a previously validated software the 16 segmental T2* values and the mean global heart T2* value were provided. A conservative cut off of 20 ms was considered the limit of normal for the segmental and global T2* values.

## Results

We identified 4 groups of patients: group I (17%) with homogeneous MIO (all segments with T2*<20 ms), group II (12%) with heterogeneous MIO (some segments with T2*<20 ms and others with T2*≥20 ms) and global heart T2*<20 ms; group III (29%) with heterogeneous MIO and global heart T2*≥20 ms; group IV (42%) with no MIO (all segments with T2*≥20 ms). The percentage of patients with cardiac complications was significantly different in the 4 groups (group I 24.6%, group II 20.6%, group III 8.4%; group IV 16.4%; P<0.0001). In particular, the percentage of patients with heart failure was significantly different in the 4 groups (group I 17.4%, group II 16.5%, group III 4.2%, group IV 8.3%; P<0.0001). No significant differences were found among groups in the percentage of arrhythmias and pulmonary hypertension. Odds Ratio for cardiac complications was 1.7 (1.0-2.7 OR 95% CI; P=.041) for patients with homogeneous MIO vs patients with no MIO. Odds Ratio for heart failure was 2.3 (1.3-4.2 OR 95% CI; P=0.004) for patients with homogeneous MIO versus patients with no MIO and 2.2 (1.1-4.2 OR 95% CI; P=.020) for patients with heterogeneous MIO and global heart T2*<20 ms versus patients with no MIO.

## Conclusions

Homogeneous MIO predicts a significantly higher risk to develop cardiac complications, especially heart failure, suggesting an intensive chelation therapy in this group of patients.

## Funding

“No-profit” support by industrial sponsorships (Chiesi, Apotex and GE Healtcare) and “Ministero della Salute, fondi ex art. 12 D.Lgs. 502/92 e s.m.i., ricerca sanitaria finalizzata anno 2006” e “Fondazione L. Giambrone”.

**Table 1:** 

	Homogeneous MIO (N=138)	Heterogeneous MIO and global Heart T2* < 20 ms (N=97)	Heterogeneous MIO and global Heart T2* ≥ 20 ms (N=238)	No MIO (N=339)	P-value
**Sex (M/F)**	67/71	38/59	119/119	157/172	0.304
**Age (years)**	28.9 ± 7.4	30.8 ± 7.4	30.4 ± 8.9	31.1 ± 8.9	0.069
**Hb pre-transfusion (g/dl)**	9.7 ± 0.6	9.6 ± 0.5	9.7 ± 0.6	9.6 ± 0.8	0.309
**Ferritin levels (ng/l)**	2454 ± 1969	2043 ± 1796	1328 ± 1277	1114 ± 1004	<0.0001
**Cardiac disease, n (%)**	34 (24.6%)	20 (20.6%)	20 (8.4%)	56 (16.5%)	<0.0001
**Heart failure, n (%)**	24 (17.4%)	16 (16.5%)	10 (4.2%)	28 (8.3%)	<0.0001
**Arrhythmias, n (%)**	13 (9.4%)	6 (6.2%)	8 (3.4%)	23 (6.8%)	0.112
**Pulmonary hypertension, n (%)**	3 (2.2%)	1 (1.0%)	4 (1.7%)	14 (4.1%)	0.192

